# Scientometric analysis of post-stroke depression research based on CiteSpace

**DOI:** 10.1097/MD.0000000000033633

**Published:** 2023-05-05

**Authors:** Saixue Tang, Mingzhou Gao, Xunshu Cheng, Lijin Ji

**Affiliations:** a Shandong University of Traditional Chinese Medicine, Jinan, Shandong Province, China; b The Affiliated Hospital of Shandong University of TCM, Jinan, Shandong Province, China; c Fujian University of Traditional Chinese Medicine, Fuzhou, Fujian Province, China.

**Keywords:** Citespace, emerging trends, knowledge domain, post-stroke depression

## Abstract

Post-stroke depression (PSD) has served as a severe and common complication leading to a higher level of mortality. Though various studies have been focused on PSD, limited research endeavor has been dedicated to bibliometric analysis in the past. In view of this, the current analysis serves to elucidate the latest status of global research and pinpoint the emerging area of interest for PSD, in order to support further investigation of the field. Publications related to PSD were retrieved from the Web of Science Core Collection database on September 24, 2022, and included in the bibliometric analysis. VOSviewer and CiteSpace software were used to visually analyze publication outputs, scientific cooperation, highly-cited references, and keywords to identify the current status and future trends in PSD research. A total of 533 publications were retrieved. The annual number of publications showed an increasing trend from 1999 to 2022. In terms of country and academic institution, the USA and Duke University have topped the list of PSD research respectively. Meanwhile, Robinson RG and Alexopoulos GS have been the most representative investigators of the field. In the past, researchers focused on the risk factors of PSD, late-life depression, and Alzheimer disease. In recent years, further research effort has been placed on meta-analysis, ischemic stroke, predictor, inflammation, mechanism, and mortality. In conclusion, in the past 20 years, PSD research has been progressing and gaining more attention. The bibliometric analysis successfully unveiled the field’s major contributing countries, institutions, and investigators. Furthermore, current hot spots and future trends in the field of PSD were identified, which included meta-analysis, ischemic stroke, predictor, inflammation, mechanism, and mortality.

## 1. Introduction

Post-stroke depression (PSD) is a common and severe sequela of stroke and a frequent affective disorder. The prevalence of the disorder ranges from 18 to 33% and varies due to study context and conditions.^[1–3]^ Post-stroke brain damage negatively impacts the patient’s mental status affecting the patients’ quality of life.^[4]^ PSD could lead to cognitive impairment in patients,^[5]^ reduce the patient’s ability to perform daily activities and self-care,^[6]^ worsen clinical outcomes of hypertension and diabetes, increase the recurrence of cardiovascular and cerebrovascular events, and increase the risk of suicide and mortality rate.^[7]^ Thus, most suffer from decreased quality of life and a heavy burden on families.

Despite the significant progress made by researchers, many questions remain to be addressed. The pathogenesis of PSD is considered complicated, involving genetic and environmental factors. Neurobiological and psychosocial mechanisms have played a central role in PSD. Dysregulation of the hypothalamic-pituitary-adrenal axis, increased inflammatory factors, decreased monoamine levels, glutamate-mediated excitotoxicity, and abnormal neurotrophic responses were previously described among PSD patients.^[8–10]^ Further, female gender, previous history of mental illness, large or multiple strokes, frontal/anterior or basal ganglia injury, history of stroke within the past year, poor social support, and significant disability are the major risk factors for PSD.^[11,12]^ Currently, both pharmacotherapy and psychotherapy are adopted to treat PSD.^[13,14]^ Meanwhile, the wide-ranging and cross-disciplinary nature of the PSD study could represent a challenge for researchers to pinpoint key information in the field.

Recently, scientometric analysis has become increasingly popular. Compared to other reviews, bibliometrics could analyze quantitatively publications to visualize the characteristics of knowledge resources and displays scientific and technical knowledge and internal connections.^[15]^ More exactly, bibliometrics measures “literature attributes and literature-related processes.”^[16]^ Its statistical scope includes authors, keywords, countries, institutions, and other elements most directly related to the literature, and through word frequency analysis, citation analysis, and co-citation analysis to reveal the research content, current research hotspots, and future research trends in a certain field over time,^[17]^ which plays a vital role in analyzing and understanding the development academic disciplines or research directions.

In this paper, we analyzed the annual frequency, journals, national institutions, co-occurrences of PSD, co-citations, and research frontiers of the relevant literature by bibliometric methods to sort out the current status and development of PSD research and to provide a reference for further research.

## 2. Methods

### 2.1. Data sources and search strategy

**Figure 1.**
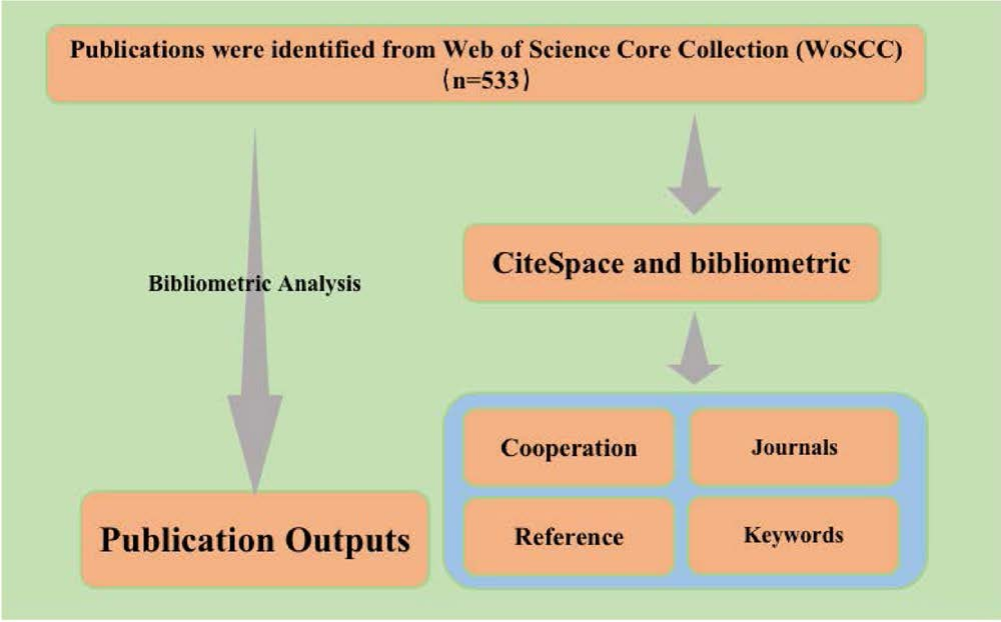
The workflow of scientometric analysis.

The data was retrieved on September 24, 2022, and collected from the Web of Science Core Collection (WoSCC). The WoSCC was adopted based on its better accuracy literature and data comprehensiveness for bibliometric analysis, compared to other databases like Scopus, Derwent, China National Knowledge Infrastructure, and Chinese Social Sciences Citation Index.“Poststroke depression,” “depression AND cerebrovascular disorders,” “vascular depression,” “stroke AND antidepressant,” and “stroke AND antidepressant AND depression” limited to Title as retrieval terms were utilized in data search for relevant studies.^[18]^ The search period was not restricted. The language was limited to English, and the category of the article was confined to article or review. The flowchart of the scientometric analysis is illustrated in Figure 1. Ethical approval was not necessary because the data do not contain any private information of patients.

### 2.2. Data extraction and collection

Literature screening and quality evaluation were carried out independently by 2 researchers. Bibliometric data, including the date of publication, the annual number of publications/citations, countries/regions, institutions, authors, journals, references, and keywords were obtained from these publications.

### 2.3. Statistical analysis

Microsoft Office Excel 2021 was used to manage data and analyze annual publications. CiteSpace is a JAVA-based citation visualization software developed by Chaomei Chen. The application allows researchers to compare existing methods and research new ideas. In the current study, CiteSpace was used for bibliometric analysis, including country, institute, keyword, category, reference, and cited journal with VOSviewer.

## 3. Results

### 3.1. Publication outputs

**Figure 2.**
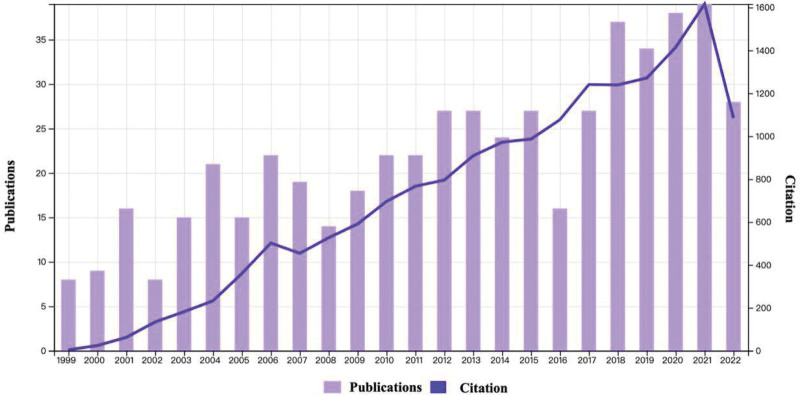
The annual number of global publications and citations from 1999 to 2022.

A total of 533 publications were retrieved. Figure 2 depicts the annual number of global publications and citations from 1999 to 2022. The annual number of publications related to PSD showed an upward trend during the period. The number of publications has increased from 8 in 1999 to 39 in 2021. Additionally, PSD literature output did decline in 2019, possibly due to coronavirus 2019. But on the whole, the increasing research output has aligned with a rising number of citations in recent years, indicating a growing academic interest in the field.

### 3.2. Cooperation map of country/region and institutes

**Figure 3.**
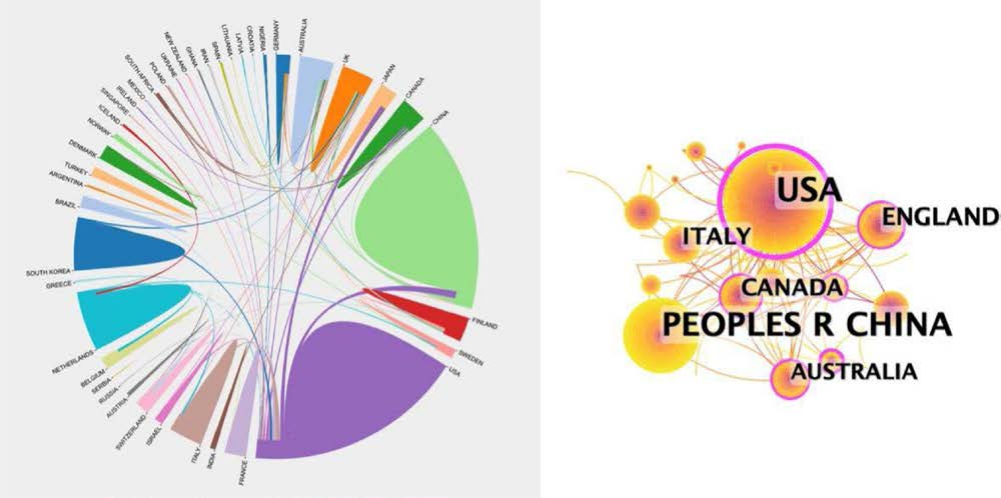
Research collaboration map between countries and regions.

Scientific cooperation could be reflected by the cooperation map of the country/region and institute via CiteSpace. From 1999 to 2022, scholars from 50 countries/areas published PSD-related research. The top 10 countries with the highest research output are listed in Table 1. Among them, the USA (n = 166, 64.9% of all papers) has published the greatest number of papers, followed by the People’s Republic of China (n = 133, 64.9% of all papers), England (n = 37, 64.9% of all papers), Canada (n = 29, 64.9% of all papers), and Italy (n = 29, 64.9% of all papers). Figure 3 shows a visualization map of collaboration analysis among countries/regions.

**Table 1:** The top 10 countries in terms of publications count and centrality.

Rank	Countries	Count	Centrality
1	USA	166	0.96
2	People’s R China	133	0.02
3	England	37	0.15
4	Canada	29	0.18
5	Italy	29	0.01
6	Australia	28	0.15
7	Netherlands	27	0
8	South Korea	25	0
9	Japan	24	0
10	Germany	15	0.16

**Figure 4.**
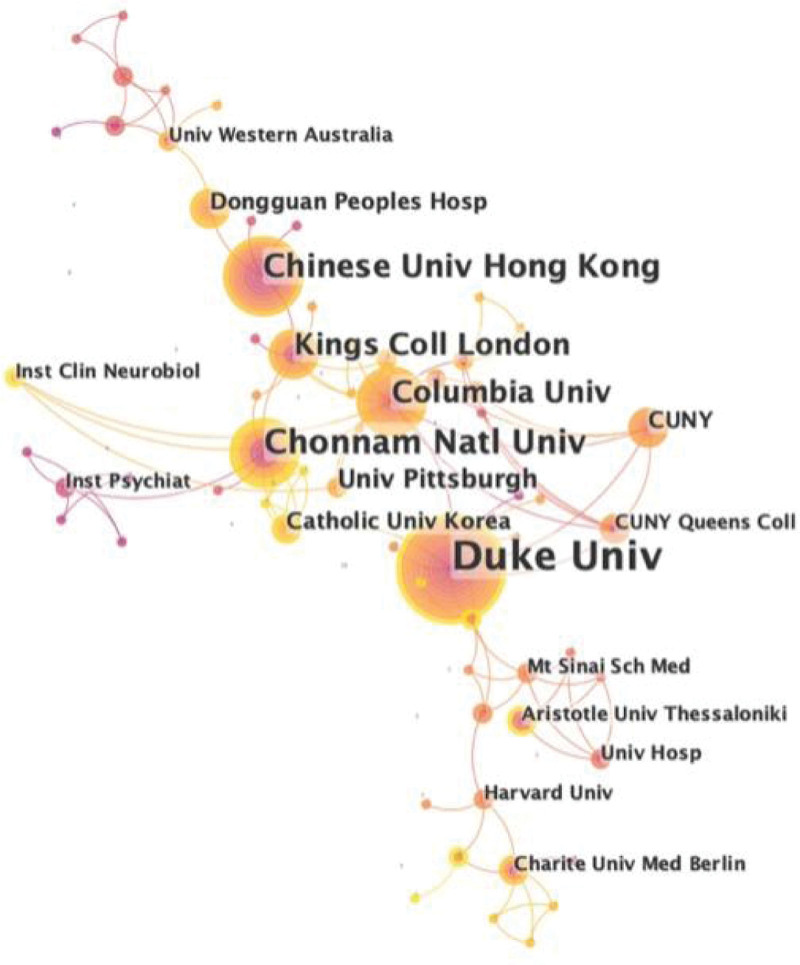
Research collaboration map between institutions.

Besides, 533 documents were contributed by 480 different institutions. Figure 4 shows the collaborations among them. Duke University, Kings College of London, and Columbia University are in larger circles on the network map, implying the importance of these institutions in the whole cooperation relationship. The top 10 institutions with the most articles are listed in Table 2. The University of Iowa ranked first with 29 papers, followed by Duke University (16 publications) and Chonnam National University (9 publications). Overall, Duke University is the key institution in this field, based on the count of research output and centrality.

**Table 2:** The top 10 institutes in terms of publication count and centrality.

Rank	Count	Institute	Rank	Centrality	Institute
1	29	Univ Iowa	1	0.02	Duke Univ
2	16	Duke Univ	2	0.02	Kings Coll London
3	9	Chonnam Natl Univ	3	0.02	Columbia Univ
4	8	Chinese Univ Hong Kong	4	0.01	Chonnam Natl Univ
5	8	IRCCS	5	0.01	Chinese Univ Hong Kong
6	7	Chang Gung Univ	6	0.01	Catholic Univ Korea
7	7	Kings Coll London	7	0.01	Dongguan Peoples Hosp
8	7	Columbia Univ	8	0.01	Univ Western Australia
9	6	Aarhus Univ Hosp	9	0.01	Harvard Univ
10	6	Chongqing Med Univ	10	0.01	Massachusetts Gen Hosp

### 3.3. Analysis of authors and cited authors

**Figure 5.**
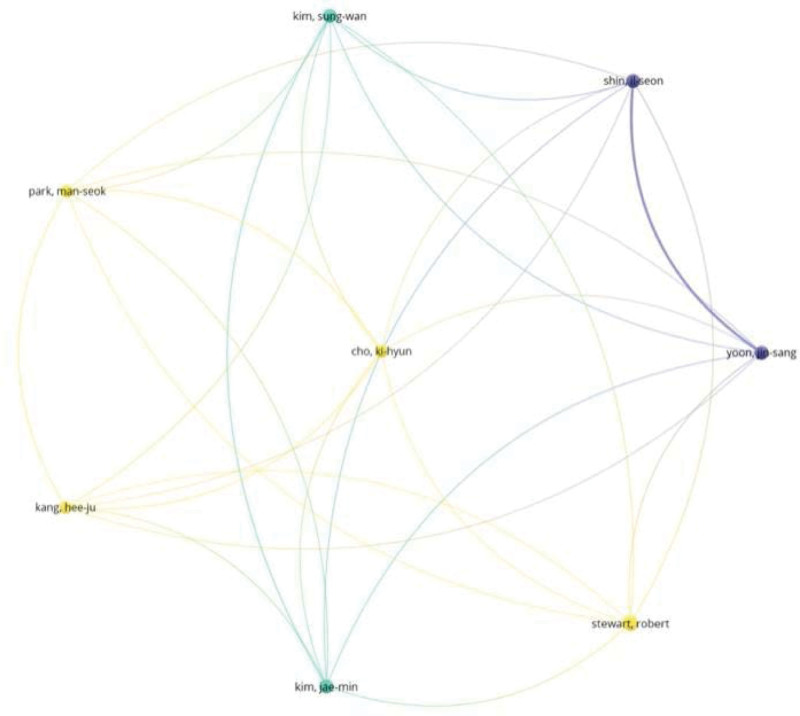
The research collaboration map of authors (biggest cooperation network).

Scientific cooperation also could be reflected by the microcosmic author cooperation network. For the number of publications, there are 4 authors whose total number of articles exceeds 10, namely Robinson RG (30 publications), Jorge RE (12 publications), Steffens DC (10 publications), and Stewart R (10 publications). Two or more authors who were cited by another or more papers at the same time constitute a co-cited relationship, and the 2 or more authors are identified as the co-cited authors. As coauthor, in terms of count, Robinson RG (198) ranked first, followed by Alecopoulos GS (152). Centrality is a parameter used to measure the importance of nodes in the network. Here, Alexopoulos GS has topped this list (Table 3). From above, Robinson RG and Alexopoulos GS are the key leaders in this field (Fig. 5).

**Table 3:** The top 10 authors and cited coauthors related to PSD.

Ranking	Author	Publications	Ranking	Count	Co-author	Ranking	Count	Co-author
1	Robinson RG	30	1	198	Robinson RG	1	0.23	Alexopoulos GS
2	Jorge RE	12	2	152	Alexopoulos GS	2	0.16	Hamilton M
3	Steffens DC	10	3	133	Hackett ML	3	0.09	Paolucci S
4	Stewart R	10	4	106	Folstein MF	4	0.09	Carson AJ
5	Zhang Y	9	5	96	Hamilton M	5	0.07	House A
6	Kim JM	8	6	81	Morris PLP	6	0.07	Williams LS
7	Chen Y	7	7	68	Krishnan KRR	7	0.06	Krishnan KRR
8	Kaste M	7	8	63	Astrom M	8	0.06	Aben I
9	Mast BT	7	9	62	Ayerbe L	9	0.06	Greenwald BS
10	Narushima K	7	10	62	Andersen G	10	0.06	Herrmann LL

PSD = post-stroke depression.

### 3.4. Journal and co-cited journal

Two hundred twenty-four journals published 533 papers on PSD. Many journals were psychiatry and neurology-related. The top 10 journals are listed by the number of publications in Table 4. The publishers of these journals were primarily located in the USA with only 3 journals’ impact factors exceeding 5. Co-cited journals were those cited together by other researchers. Table 5 presents the top 5 cited journals with the highest frequency of PSD. The most frequently cited journal was *Stroke*, followed by the *American Journal of Psychiatry and Biological Psychiatry*.

**Table 4:** The top 10 journals with the highest frequency of PSD.

Ranking	Journal	Research direction	Count	IF 2021	Country
1	*International Journal of Geriatric Psychiatry*	Psychiatry	30	3.85	England
2	*American Journal of Geriatric Psychiatry*	Psychiatry	28	7.996	USA
3	*Stroke*	Clinical Neurology	26	10.17	USA
4	*Journal of Affective Disorders*	Clinical Neurology	22	6.533	USA
5	*Journal of Stroke Cerebrovascular Diseases*	Surgery	17	2.677	USA
6	*Medicine*	Internal Medicine	15	1.817	USA
7	*Journal of Neuropsychiatry and Clinical Neurosciences*	Psychiatry	10	2.891	USA
8	*Cerebrovascular Diseases*	Clinical Neurology	9	3.104	USA
9	*Evidence Based Complementary and Alternative Medicine*	General Practice and Complementary Medicine	9	2.65	England
10	*Archives of Physical Medicine and Rehabilitation*	Rehabilitation Medicine	8	4.06	USA

IF = impact factor, PSD = post-stroke depression.

**Table 5:** The top 10 cited journals with the highest frequency of PSD.

Ranking	Count	Centrality	Co-cited journal	IF 2021	Country
1	397	0.01	*Stroke*	10.17	USA
2	313	0.01	*American Journal of Psychiatry*	19.242	USA
3	260	0.01	*Biological Psychiatry*	12.810	USA
4	257	0.02	*Archives of General Psychiatry*	–	USA
5	238	0.01	*British Journal of Psychiatry*	10.671	England
6	232	0	*Journal of Neurology Neurosurgery and Psychiatry*	13.654	England
7	210	0.01	*Journal of Affective Disorders*	6.533	USA
8	196	0.01	*Lancet*	202.731	England
9	194	0.01	*American Journal of Geriatric Psychiatry*	7.996	USA
10	181	0.04	*International Journal of Geriatric Psychiatry*	3.85	England

IF = impact factor, PSD = post-stroke depression.

### 3.5. Highly-cited publications and co-cited references

Highly-cited publications and co-cited references were fundamental and the basis of a particular field. In this study, the top 10 cited publications are shown in Table 6. Eight publications were cited >200 times. The article (entitled Late-life depression and risk of vascular dementia and Alzheimer’s disease: systematic review and meta-analysis of community-based cohort studies) published in 2013 by Diniz, Breno S et al obtained the largest number of citations (666).

**Table 6:** The top 10 highly cited studies of PSD.

Title	Authors	Source title	Publication year	Total citations
Late-life depression and risk of vascular dementia and Alzheimer disease: systematic review and meta-analysis of community-based cohort studies	Diniz, Breno S.	*British Journal of Psychiatry*	2013	666
The vascular depression hypothesis: mechanisms linking vascular disease with depression	Taylor, W. D.	*Molecular Psychiatry*	2013	460
Poststroke depression: Prevalence, diagnosis, treatment, and disease progression	Robinson, RG	*Biological Psychiatry*	2003	317
Poststroke depression correlates with cognitive impairment and neurological deficits	Kauhanen, ML	*Stroke*	1999	295
Poststroke Depression A Scientific Statement for Healthcare Professionals From the American Heart Association/American Stroke Association	Towfighi, Amytis	*Stroke*	2017	257
Escitalopram and problem-solving therapy for prevention of poststroke depression - A randomized controlled trial	Robinson, Robert G.	*Jama-journal of the American Medical Association*	2008	244
Improved recovery in activities of daily living associated with remission of poststroke depression	Chemerinski, E	*Stroke*	2001	206
Poststroke depression - An 18-month follow-up	Berg, A	*Stroke*	2003	205
Mortality and poststroke depression: A placebo-controlled trial of antidepressants	Jorge, RE	*American Journal of Psychiatry*	2003	196
Support for the Vascular Depression Hypothesis in Late-Life Depression Results of a 2-Site, Prospective, Antidepressant Treatment Trial	Sheline, Yvette I.	*Archives of General Psychiatry*	2010	194

PSD = post-stroke depression.

**Figure 6.**
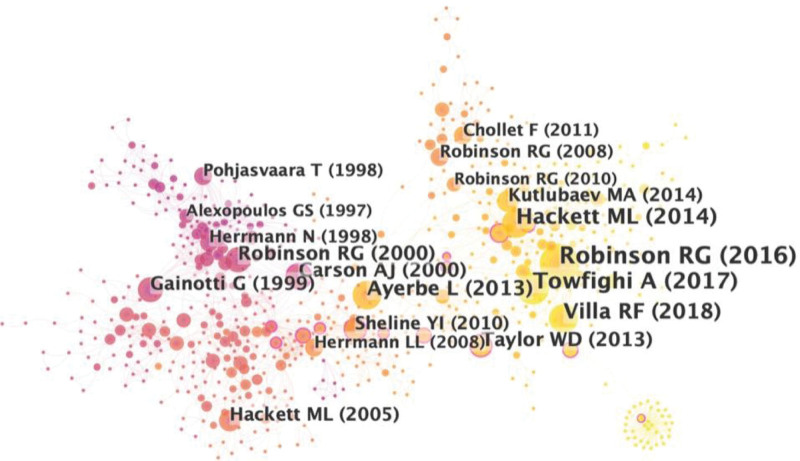
The research collaboration map of co-reference.

Next, an analysis for all the co-cited references by CiteSpace was constructed, as shown in Figure 6. The top 10 cited references about frequency and centrality are shown in Tables 7 and 8. According to the ranking of frequency and centrality in cited references, review papers were found to be the major category of publication. Only a few original research papers were used. The most frequently cited article was published in *American Journal of Psychiatry* titled, “Post-Stroke Depression: A Review.”^[18]^ In this review, relevant etiological factors such as Inflammatory processes, genetic and epigenetic variations, white matter disease, cerebrovascular deregulation, altered neuroplasticity, and changes in glutamate neurotransmission have also been discussed for an understanding of the PSD pathophysiology. Besides, the article published in *STROKE*, titled “Natural History, Predictors, and Associations of Depression 5 Years After Stroke,” was the highest-ranked citation in terms of centrality. This article estimated the frequency, predictors, and associations of depression up to 5 years after stroke in a population-based study showing us the longer-term natural history of depression after stroke.^[19]^

**Table 7:** The top 10 cited references for the highest frequency of PSD.

Ranking	Frequency	Title	Author	Source
1	34	Post-stroke depression: a review	Robinson RG	*AM J PSYCHIAT*
2	28	Poststroke depression: a scientific statement for healthcare professionals from the American Heart Association/American Stroke Association	Towfighi A	*STROKE*
3	25	Part I: frequency of depression after stroke: an updated systematic review and meta-analysis of observational studies	Hackett ML	*INT J STROKE*
4	23	Post-stroke depression: mechanisms and pharmacological treatment	Villa RF	*PHARMACOL THERAPEUT*
5	21	Natural history, predictors and outcomes of depression after stroke: systematic review and meta-analysis	Ayerbe L	*BRIT J PSYCHIAT*
6	17	Depression after stroke and lesion location: a systematic review	Carson AJ	*LANCET*
7	17	The vascular depression hypothesis: mechanisms linking vascular disease with depression	Taylor WD	*MOL PSYCHIATR*
8	17	Nortriptyline versus fluoxetine in the treatment of depression and in short-term recovery after stroke: a placebo-controlled, double-blind study	Robinson RG	*AM J PSYCHIAT*
9	15	Frequency, phenomenology and anatomical – clinical correlates of major post-stroke depression	Gainotti G	*BRIT J PSYCHIAT*
10	14	Predictors of depression after stroke: a systematic review of observational studies	Hackett ML	*STROKE*

PSD = post-stroke depression.

**Table 8:** The top 10 cited references for the highest centrality of PSD.

Ranking	Centrality	Title	Author	Source
1	0.19	Natural History, Predictors, and Associations of Depression 5 Years After Stroke	Ayerbe L	*STROKE*
2	0.18	Depression after stroke and lesion location: a systematic review	Carson AJ	*LANCET*
3	0.17	Focal or generalized vascular brain damage and vulnerability to depression after stroke: a 1-year prospective follow-up study	Aben I	*INT PSYCHOGERIATR*
4	0.15	fMRI Correlates of White Matter Hyperintensities in Late-Life Depression	Aizenstein HJ	*AM J PSYCHIAT*
5	0.14	Post-stroke depression: mechanisms, translation and therapy	Loubinoux I	*J CELL MOL MED*
6	0.13	Vascular depression consensus report—a critical update	Aizenstein HJ	*BMC MED*
7	0.13	Microstructural White Matter Abnormalities and Remission of Geriatric Depression	Alexopoulos GS	*AM J PSYCHIAT*
8	0.12	Clinical Presentation and Outcome of Geriatric Depression in Subcortical Ischemic Vascular Disease	Bella R	*GERONTOLOGY*
9	0.12	Mechanisms and treatment of late-life depression	Alexopoulos GS	*TRANSL PSYCHIAT*
10	0.11	Support for the Vascular Depression Hypothesis in Late-Life Depression	Sheline YI	*ARCH GEN PSYCHIAT*

PSD = post-stroke depression.

### 3.6. Keywords co-occurrence and burst analysis

**Figure 7.**
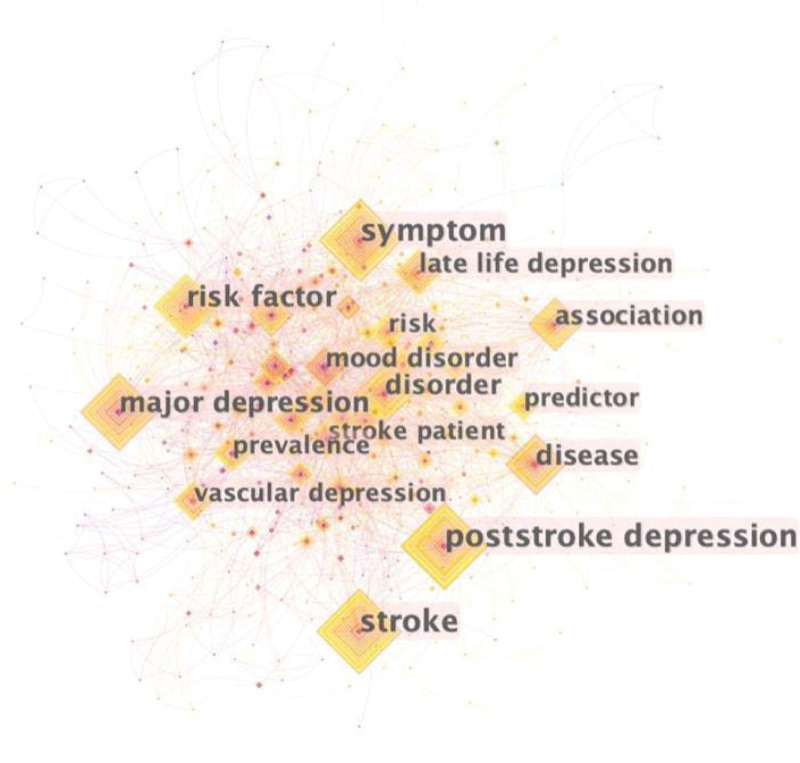
The map of keyword co-occurrence in literatures.

To investigate the research hotspots in the field of PSD, a total of 493 keywords were extracted from the 533 publications for co-occurrence analysis on CiteSpace software, as shown in Figure 7. Table 9 shows the top 10 highest-frequency and highest-centrality keywords. Among these keywords, “poststroke depression” (101 times) frequently appeared, followed by “symptom” (98 times), “stroke” (96 times), “major depression” (72 times), and “risk factor” (67 times). In terms of centrality, “Alzheimer’s disease” (0.15) was the most central, followed by “major depression” (0.14), “disease” (0.14), “symptom” (0.11), and “disorder” (0.11).

**Table 9:** The top 10 keywords for the highest count and centrality of PSD.

Rank	Count	Keywords	Rank	Centrality	Keywords
1	101	Poststroke depression	1	0.15	Alzheimer disease
2	98	Symptom	2	0.14	Major Depression
3	96	Stroke	3	0.14	Disease
4	72	Major depression	4	0.11	Symptom
5	67	Risk factor	5	0.11	Disorder
6	56	Disease	6	0.11	Association
7	53	Disorder	7	0.11	Impairment
8	47	Late life depression	8	0.1	Poststroke depression
9	45	Mood disorder	9	0.1	Stroke
10	44	Association	10	0.1	Follow up

PSD = post-stroke depression.

**Figure 8.**
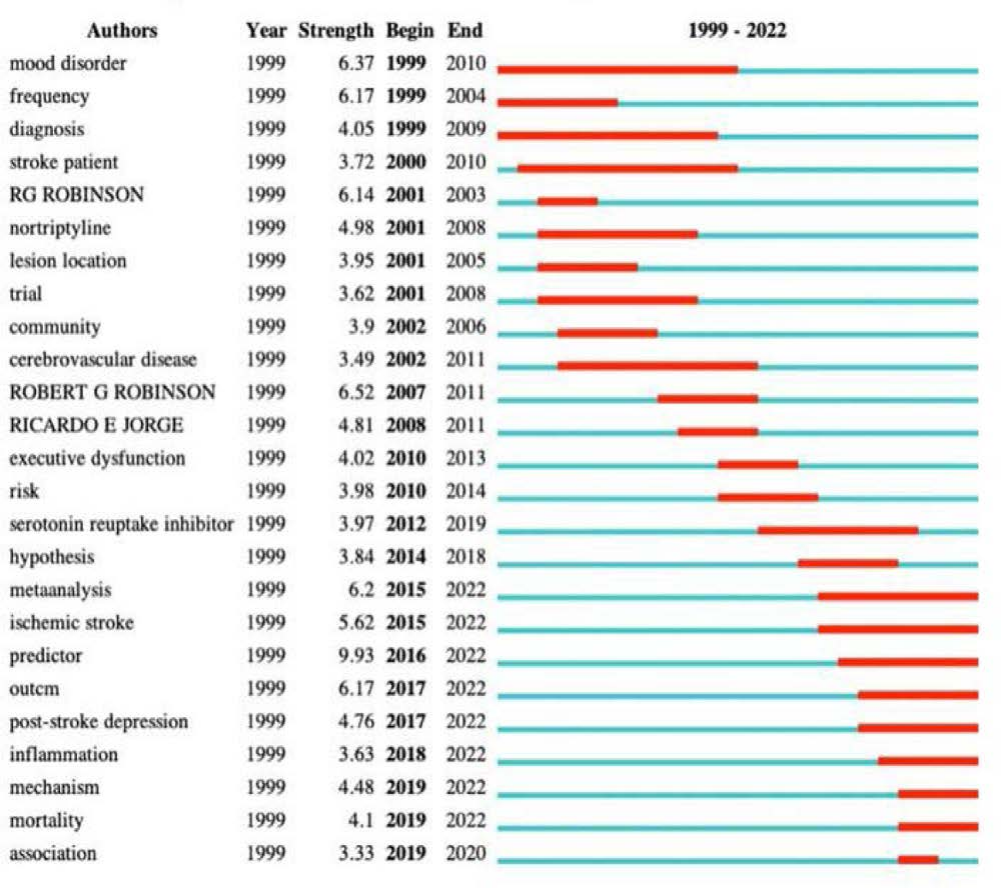
The top keywords with the strongest citation bursts.

“Burst words” mean that words are cited frequently over a period of time. The frontier of research trend could be predicted according to the distribution of keywords with the strongest citation burst. The top 25 keywords with the strongest citation burst from 1999 to 2022 are shown in Figure 8. The red bars demonstrated that the keyword was cited frequently, and the green bars showed that the keyword was cited infrequently. Meta-analysis, ischemic stroke, predictor, outcome, inflammation, mechanism, and mortality would be potentially cited frequently over the coming years, which indicates the emerging interest in the PSD study field.

## 4. Discussion

### 4.1. General information

Psychiatrists have recognized PSD for >100 years.^[9]^ To our knowledge, this is the first report analyzing both the quality and quantity of PSD. Bibliometric analysis suggested that the number of related publications increased from 1999 to 2022. This phenomenon means that PSD research has gradually attracted researchers’ attention. Focusing on research collaboration, the cooperation status from maps of the country/region, institute, and authors via CiteSpace was explored. Of 50 contributing countries, the USA has the highest level of research output, followed by the People’s Republic of China. The People’s Republic of China developed PSD guidelines and provide suggestions for clinicians and related workers.^[20]^ It can be seen that the USA has occupied the dominant position, in terms of the number of original studies and level of research collaboration.

Furthermore, 480 different institutions from 50 countries contributed 533 documents. Duke University, Kings College London, and Columbia University are in larger circles on the network map, implying the higher importance of these institutions, in terms of PSD research collaboration. Among the institutions, Duke University has topped the list.

In terms of authorship, Robinson RG was the most prolific author with 30 publications, followed by Jorge RE (12 publications), Steffens DC (10 publications), and Stewart R (10 publications). Regarding co-cited authors, Robinson RG and Alexopoulos GS took the core position in this field. Besides, journal analysis can help follow-up researchers quickly identify the core journals in this research field. Five hundred thirty-three papers on PSD come from 224 journals. Most of the articles are psychiatry and neurology-related. *International Journal of Geriatric Psychiatry* is the leading journal, in terms of publication number.

On the other hand, we also explored the co-cited journals. *International Journal of Geriatric Psychiatry* and *Stroke* are identified as the primary source of frequently cited publications. Among the 533 publications, the article (entitled Late-life depression and risk of vascular dementia and Alzheimer’s disease: systematic review and meta-analysis of community-based cohort studies) published in 2013 by Diniz, Breno S et al obtained the largest number of citations (666). Next, the high level of frequency and centrality in cited references suggested that “Post-Stroke Depression: A Review” may be influential towards the follow-up research in the PSD field.

### 4.2. Hotspots and frontiers

Regarding current hotspots and frontiers in the future, keyword co-occurrence and burst analysis were adopted for analysis. Results showed that current research hotspots include risk factors, late-life depression, and Alzheimer disease. PSD is the most frequent neuropsychiatric condition after a stroke and is heterogenous in nature.^[21]^ Most studies believe PSD or neuropsychiatric condition is closely related to physiological, psychological, social, and lifestyle factors.^[22–25]^ Previous studies also suggested that PSD is associated with a myriad of factors, such as age,^[26]^ sex differences (greater hazard of developing PSD in females),^[27]^ and education level,^[28]^ but the results appeared to be inconsistent from time to time. Besides, social-psychological factors take part in the occurrence of PSD, such as stressful life events,^[29]^ health education, social support,^[30]^ premorbid neuroticism,^[31]^ insomnia, and the degree of neurological deficit.^[32]^ Abnormal physiological conditions, including the dysregulation of inflammatory factors and neurotrophic factors, could potentially contribute to the onset of depression. For instance, a significant reduction of brain-derived neurotrophic factor expression could be seen among patients who experienced strokes. The alternation of gene expression would contribute to the development of depression.^[10]^ And there is a significant association between right hemisphere stroke and the incidence of depression for studies with subacute post-stroke phase.^[33]^ It is expected that further research focusing on PSD risk factors would be made available.

Besides, late-life depression and Alzheimer disease have attracted researchers’ attention, due to their close relationship with PSD. Established studies indicated that the severity of asthenia and emotional disorders in post-stroke patients increased.^[34]^ Reaffirmed by previous literature, stroke is a common and frequently occurring disease affecting human health and quality of life. Most of the patients are middle-aged and elderly people.^[35]^ Understandably, there is a close relationship between the post-stroke effect and the onset of Alzheimer disease in the elderly due to cognitive impairment.^[36,37]^ Late-life depression is becoming prevalent, with an estimated 15% prevalence among people 65 years of age.^[38]^ Disease preventive measures and after-stroke care have been an area of focus for the investigator.

Meta-analysis, ischemic stroke, predictor, inflammation, mechanism, and mortality represent the emerging trends in the PSD study field.

### 4.3. Meta-analysis

Meta-analysis is a robust methodology of analysis combining published and unpublished data with growing popularity.^[39]^ In recent years, a number of publications have been focused on PSD adopting meta-analysis. For example, Shi Y et al has successfully revealed a myriad of risk factors associated with PSD.^[40]^; Li LJ et al examined the efficacy and safety of paroxetine in treating PSD in the future, based on 212 patients^[41]^; Wang SB et al investigated cognitive behavioral therapy for PSD via meta-analysis^[42]^; also, Bartoli F et al explored the association between early PSD and mortality to show the negative impact of early PSD on patient survival rates.^[43]^ In the future, more meta-analysis is expected to be made available in the PSD field.

### 4.4. Ischemic stroke

Stroke, the most common form of cerebrovascular disease, is a leading cause of death and disability worldwide. Stroke is broadly classified into ischemic and hemorrhagic categories.^[44]^ In the US, 87% and 13% of stroke diagnoses are ischemic and hemorrhagic respectively.^[45]^ As an acute and severe cerebral vascular disease, depression after an ischemic cerebrovascular accident is a frequent phenomenon occurring in 30 to 50% of patients.^[46]^ The disease burden has adversely impacted people’s health and brought about substantial economic burdens to society.^[47]^

### 4.5. Predictor

Identifying factors associated with PSD is very important due to the underdiagnosis and undertreatment of PSD. At present, various studies indicated that chronic stress (including financial and health-related stress, irrespective of age),^[48]^ IL-33 concentration of ≤71.85 ng/L^[49]^ significantly increased the risk for PSD. And Shi YZ et al pointed out that female gender, smoking, mild global cognitive impairment, and stroke recurrence predict early-onset or late-onset PSD after minor ischemic stroke.^[50]^ In Brazil, depressive symptoms are common among elderly survivors after stroke, and the degree of functional dependency could serve as the main predictor of PSD.^[51]^ Recent studies indicated that trunk control and basic activities of daily living are vital for preventing the consequences of PSD.^[52]^ Identifying more predictors of PSD is extremely important for disease prevention and diagnosis in the future.

### 4.6. Inflammation

According to inflammation theory, PSD may be related to the death of inflammatory binding cells in brain regions involved in emotional response, caused by an overactivated inflammatory response. The stroke will aggravate cerebrovascular dysfunction, leading to the emergence of brain inflammation and the death of nerve cells.^[53]^ Cytokines (IL-1, IL-2, IL-6, TNF- α) could promote the gene expression of indoleamine 2,3-dioxygenase, metabolize tryptophan into canine uric acid, transfer tryptophan from 5-HT synthesis, reduce the content of 5-HT in the frontal cortex and basal ganglia.^[54]^ The physiological change could contribute to the onset of depression. Some studies have also shown that the peripheral immune inflammatory reaction caused by acute stroke is related to PSD, but the exact mechanism has yet to be unveiled.

### 4.7. Mortality

Stroke is the leading cause of long-term disability and the world’s second leading cause of death. The mortality of stroke patients with depression is significantly higher than those without depression.^[55]^ Possible causes of increased mortality in PSD patients are also the focus of the study. Ayerbe et al showed that PSD subjects showed higher mortality within 5 years after stroke.^[56]^ Bartoli et al evaluated the association between early PSD and mortality and found that the mortality of PSD subjects was higher than those without PSD.^[43]^ Currently, there is a stronger association between depression and mortality in patients over 65 years of age.^[56]^ Furthermore, depression increases the risk of cardiovascular disease, which may lead to recurrent cerebrovascular events and higher mortality.^[57]^ Identifying the possible causes of increased mortality in PSD patients is particularly important for patients’ treatment and recovery.

## 5. Strengths and limitations

This is pioneering research on visual analysis in the PSD field based on the bibliometric approach to understanding the trends and hotspots. However, due to the limitations of the currently used scientometric software, this study only used the WoSCC database for screening the literature, which may have the possibility of relevant literature not being included. Future analyses will make use of more databases.

## 6. Conclusions

We summarized the 533 articles from 1999 to 2022 in the field of PSD to identify the current status and global trends. Our bibliometric analysis shows that the attention in this field continues to rise. The USA has occupied the dominant position and collaborated with other countries. Robinson RG and Alexopoulos GS are the key leaders in the field. *International Journal of Geriatric Psychiatry* and *Stroke* is the primary source of frequently cited publications. Our results also support future research. The meta-analysis, ischemic stroke, predictor, inflammation, mechanism, and mortality represent the emerging trends that are still the focus of research.

## Author contributions

**Conceptualization:** Saixue Tang.

Data curation: Saixue Tang.

Formal analysis: Saixue Tang.

Funding acquisition: Lijin Ji.

Methodology: Mingzhou Gao.

Project administration: Xunshu Cheng.

Resources: Saixue Tang.

Validation: Saixue Tang.

Visualization: Xunshu Cheng.

Writing – original draft: Saixue Tang, Mingzhou Gao.

Writing – review & editing: Mingzhou Gao.
